# Flavacol and Its Novel Derivative 3-*β*-Hydroxy Flavacol from *Streptomyces* sp. Pv 4-95 after the Expression of Heterologous AdpA

**DOI:** 10.3390/microorganisms10122335

**Published:** 2022-11-25

**Authors:** Stepan Tistechok, Marc Stierhof, Anna Kachor, Maksym Myronovskyi, Oleksandr Gromyko, Andriy Luzhetskyy

**Affiliations:** 1 Department of Genetics and Biotechnology, Ivan Franko National University of Lviv, 79005 Lviv, Ukraine; 2 Department of Pharmaceutical Biotechnology, Saarland University, 66123 Saarbruecken, Germany; 3 Explogen LLC, 79005 Lviv, Ukraine; 4 Microbial Culture Collection of Antibiotic Producers, Ivan Franko National University of Lviv, 79005 Lviv, Ukraine; 5 Helmholtz Institute for Pharmaceutical Research Saarland, 66123 Saarbruecken, Germany

**Keywords:** flavacol, 3-*β*-hydroxy flavacol, AdpA, secondary metabolites, *Streptomyces*

## Abstract

Actinomycetes are one of the main producers of biologically active compounds. However, their capabilities have not been fully evaluated due to the presence of many unexpressed silent clusters; moreover, actinomycetes can probably produce new or previously discovered natural products under certain conditions. Overexpressing the *adpA* gene into streptomycetes strains can unlock silent biosynthetic gene clusters. Herein, we showed that by applying this approach to *Streptomyces* sp. Pv 4-95 isolated from *Phyllostachys viridiglaucescens* rhizosphere soil, two new mass peaks were identified. NMR structure analysis identified these compounds as flavacol and a new 3-*β*-hydroxy flavacol derivative. We suggest that the presence of heterologous AdpA has no direct effect on the synthesis of flavacol and its derivatives in the Pv 4-95 strain. However, AdpA affects the synthesis of precursors by increasing their quantity, which then condenses into the resulting compounds.

## 1. Introduction

Actinomycetes are the most promising group of microorganisms that produce biologically active compounds [1]. These bacteria are the source of more than 70% of all microorganism-derived bioactive compounds, among which a single genus, *Streptomyces,* produces about 55% [2]. However, the ability of actinomycetes to produce biologically active compounds has long been underestimated due to the presence of biosynthetic gene clusters (BGCs), which code for new natural products that are not expressed or are expressed at low levels under standard conditions. Additionally, when using traditional methods to screen actinomycetes for producers of new compounds that exhibit antibiotic activity, a significant part of natural strains are biologically inactive, so they may not be subject to further research [3]. Activating the expression of silent BGCs in these strains can lead to the discovery of new biologically active compounds [4]. This can be involved in the deletion of inhibitory elements (repressors or their binding sites) or the insertion of activating regulatory elements [5]. One of the elements that can be used to activate silent BGCs is AdpA (the AraC–XylS family of transcriptional regulators), which controls the expression of a significant number of genes [6,7]. AdpA is also involved in the formation of aerial mycelium in streptomycetes [8]. The presence of AdpA in streptomycetes activates the biosynthesis of bioactive compounds and other microbial metabolites [7,9,10]. Manipulation of this global regulator has led to the activation of the production of an unusual angucyclinone, oviedomycin, in *Streptomyces ansochromogenes* [11]. Moreover, the recent work of our colleagues showed that overexpressing heterologous AdpA into *S. cyanogenus* S136 can activate the silent BGC of the polyene antibiotic lucensomycin [12]. Thus, utilizing this global metabolic regulator can activate the synthesis of compounds that are not synthesized under standard cultivation conditions.

In the present study, we demonstrate the activation of the production of flavacol and a new 3-*β*-hydroxy flavacol derivative by integrating the pleiotropic transcriptional regulator AdpA in the *Streptomyces* sp. Pv 4-95 strain.

## 2. Materials and Methods

### 2.1. Bacterial Strains, Plasmids and Growth Conditions

The Pv 4-95 strain that was isolated from the rhizosphere of *Phyllostachys viridiglaucescens* (Carrière) Rivière & C. Rivière (Crimean Peninsula, Ukraine) was used in this study. This strain and its recombinant strains were grown under standard conditions [13] using oatmeal (20.0 g oatmeal, 20.0 g agar, and tap water–1.0 L; pH 7.2) and liquid tryptic soy broth media (TSB, Sigma-Aldrich; Burlington, MA, United States). SG medium (20.0 g/L glucose, 10.0 g/L soy peptone, and 2.0 g/L CaCO3; pH 7.2) was used to produce the secondary metabolites. The bacterial strains were grown in Luria agar (LA; 10.0 g/L tryptone, 10.0 g/L NaCl, 5.0 g/L yeast extract and 15.0 g/L agar).

The *Escherichia coli* donor strain WM6026 [14] was used for intergeneric conjugation and was grown at 37 °C in liquid Luria broth medium (LB; 10.0 g/L tryptone, 10.0 g/L NaCl, 5.0 g/L yeast extract) containing 0.1 mM m-diaminopimelate (DAP). The antibiotic apramycin (50 μg/mL) was added as needed. The bacterial test strains *Bacillus subtilis* ATCC31324 and *Staphylococcus aureus* ATCC 25923 were used to test the antibacterial activity.

All strains were deposited in the Culture Collection of Microorganisms–Producers of Antibiotics (CCMPA) of Ivan Franko National University of Lviv (LNU).

### 2.2. DNA Extraction, Amplification and Sequencing

For total DNA isolation, strains were cultivated in TSB medium for 3 days at 28 °C and shaken at 180 rpm. Total DNA was isolated by the salting-out procedure, as described in [13]. Amplification of the 16S rRNA gene was carried out using the primers 8F (5′-AGAGTTTGATYMTGGCTCAG-3′) and 1510R (5′-TACGGYTACCTTGTTACGACTT-3′). A polymerase chain reaction (PCR) was carried out in a total volume of 50 μL containing 2.0 μL of genomic DNA (~50 ng), 0.5 μL of each primer (100 pmol), 1.0 μL of deoxynucleotide triphosphates (10.0 mM each), 5.0 μL of 10 × PCR buffer, 0.5 μL of DNA polymerase (1 U/μL), 2.5 μL dimethyl sulfoxide and 38.0 μL of Milli-Q grade water. The PCR parameters were initial denaturation at 95 °C for 5 min, followed by 30 cycles of denaturation at 95 °C for 30 s, annealing for primers at 53 °C for 30 s and extension at 72 °C for 90 s. A final extension was carried out at 72 °C for 10 min. The received PCR products were visualized in 1% agarose gel and then purified using the QIAquick Gel Extraction Kit (Qiagen, Venlo, Netherlands) and sequenced with forward and reverse primers by Explogen LLC (Lviv, Ukraine). The 16S rRNA gene sequence of *Streptomyces* sp. Pv 4-95 was deposited in GenBank with the accession number OM763959.

### 2.3. Phylogenetic Analysis of the Pv 4-95 Strain

The phylogenetic analysis of the 16S rRNA gene sequence of strain Pv 4-95 was performed using RDP Release 11 [15]. The closest 16S rRNA-related *Streptomyces* species were determined from BLAST searches in the National Center for Biotechnology Information database (https://blast.ncbi.nlm.nih.gov/Blast.cgi) and were obtained from GenBank using the Multiple Sequence Comparison by Log-Expectation (MUSCLE) alignment tool [16]. The phylogenetic tree was constructed in the Molecular Evolutionary Genetics Analysis program (MEGA X) [17] using the two-making algorithm neighbour-joining (NJ) [18]. The 16S rRNA gene sequence of *Saccharopolyspora erythrea* NRRL 2338 was used as an outgroup. The evolutionary distances were computed using the Kimura 2-parameter method [19], and the robustness of the tree topology was evaluated by a bootstrap test (1000 replicates) [20].

### 2.4. Construction of the Pv 4-95 Strain with AdpA Expression

The pTESadpA vector used for the expression of the heterologous adpA gene was provided by Dr. Ostash B. (LNU, Lviv, Ukraine). The empty vector pTES was used as a negative control. Both vectors were transferred into the strain Pv 4-95 by conjugation with *E. coli* WM6026. For conjugation, spore suspensions of Pv 4-95 strain were collected using sterile water and filtered through non-adsorbent cotton wool as described previously [21]. The resulting transconjugate colonies were checked by PCR using the primers adpa_chkF (5′-ATCGCCTCCAGCCCGTGTGG-3′) and adpa_chkR (5′-GCGTGGGTCGGTGACGTTCC-3′) for the presence of the heterologous *adpA* gene. A PCR was carried out in a total volume of 50 μL as described above.

### 2.5. Secondary Metabolite Extraction and Analysis

To extract the secondary metabolites, streptomycetes strains were grown in 15 mL of TSB in a 100 mL flask for 2 days, and 1 mL of preculture was inoculated into 100 mL of production medium in a 500 mL flask. The strains were grown for 7 days at 28 °C and 180 rpm in an Infors multitron shaker (Infors AG, Basel, Switzerland). After cultivation, the secondary metabolites were extracted with an ethyl acetate and acetone:methanol (1:1) mixture. The obtained extracts were evaporated using an IKA RV-8 rotary evaporator (IKA, Staufen, Germany) at 40 °C and dissolved in 1 mL of methanol. The extracts were analysed on a Dionex Ultimate 3000 UPLC system (ThermoFisher Scientific, Waltham, MA, USA) coupled to a PDA detector using a 100 mm ACQUITY UPLC BEH C_18_ 1.7 μm column (Waters Corporation, Milford, MA, USA). The extracts were separated by a linear gradient (from 5% to 95%) of water + 0.1% formic acid (A) and acetonitrile + 0.1% formic acid (B) as the mobile phase at a flow rate of 0.6 mL/min for 18 min. Mass analysis was performed on Bruker Amazon Speed (Bruker, Billerica, MA, USA) and Thermo LTQ Orbitrap XL (ThermoFisher Scientific, Waltham, MA, USA) mass spectrometers using the positive mode of ionization and a range detection of 200–2000 *m*/*z*. Data were analysed using Compass Data Analysis v. 4.2 (Bruker) and Xcalibur v. 3.0 (ThermoFisher Scientific).

### 2.6. Secondary Metabolite Purification

To purify the targeted compounds, the strain *Streptomyces* sp. Pv 4-95adpA was cultivated in 10 L SG (100 flasks with 100 mL of SG medium) as described above. After cultivation, the supernatant was separated from the biomass and extracted with the same amount of ethyl acetate. The obtained ethyl acetate extract was dissolved in methanol and purified in three stages. The first purification stage was normal-phase chromatography on a silica gel column with hexane (solvent A), chloroform (solvent B), ethyl acetate (solvent C) and methanol (solvent D) as the mobile phase at a flow rate of 100 mL/min. A triple linear gradient of each solvent pair A/B (15 column volumes (CV)), B/C (15 CV) and C/D (15 CV) was used, and fractions were collected every 18 mL. The separation was performed on a Biotage Isolera One LC-system (Biotage, Uppsala, Sweden). The pooled and concentrated fractions containing the compound of interest were further purified by size-exclusion chromatography on a Sephadex LH-20 column (Sigma-Aldrich, Louis, MO, USA) with methanol as the mobile phase. The fractions containing the compound of interest were again pooled together and concentrated. The last purification stage was reversed-phase high-performance liquid chromatography (HPLC), separation on a semipreparative C_18_ column SynergiTM 4 μm Fusion-RP 80 Å 250 × 10 (Phenomenex, Torrance, CA, USA) using water + 0.1% formic acid (A) and acetonitrile + 0.1% formic acid (B) as a mobile phase and a linear gradient (5% to 95%) of solvent B at a flow rate of 4 mL/min for 18 min. The fractions containing pure compounds were pooled together and evaporated. A quality assessment of the purification at each stage was verified by HPLC-MS.

### 2.7. Nuclear Magnetic Resonance Spectroscopy (NMR)

NMR spectra were recorded on a Bruker Avance 500 spectrometer (Bruker, BioSpin GmbH, Rheinstetten, Germany) equipped with a 5 mm BBO probe at 298 K. All compounds were measured in deuterated methanol (Deutero, Kastellaun, Germany). The chemical shifts are reported in parts per million (ppm) relative to TMS. All spectra were recorded using the standard pulse programs from TOPSPIN v.4.0.6 software.

## 3. Results and Discussion

### 3.1. Activation of Secondary Metabolite Production in the Pv 4-95 Strain

Actinomycetes from the plant rhizosphere are a great source for screening new natural products. Our previous studies of plant rhizosphere actinomycetes revealed producers of new natural products, such as the sesquiterpene albaflavenol B [22], the anthraquinone rubimicinone A [23], the macrolid kendomycin E [24], and many others. However, these secondary metabolites cannot always be detected using standard culture conditions, often because they are not produced, or their synthesis is below detection limits. To date, many methods have been developed to activate the production of secondary metabolites, such as the use of global regulators such as AdpA [25]. Activating silent secondary metabolite production can potentiate the discovery of new compounds. Thus, strains that did not produce in the preliminary screening can be considered potential sources of new natural compounds.

**Figure 1:**
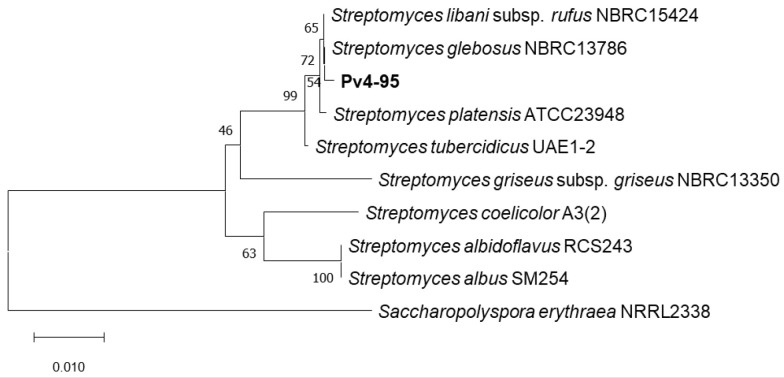
NJ tree based on 16S rRNA gene sequences from strain Pv 4-95 (in bold), their closest neighbours and several representative *Streptomyces*-type strains. *Saccharopolyspora erythraea* NRRL 2338 was used as an outgroup. Bar, 0.01 substitutions per nucleotide position.

In this study, we focused on the Pv 4-95 strain, which produced no obvious secondary metabolites under standard cultivation conditions. In addition, this strain showed no antimicrobial activity against a wide range of test cultures. Therefore, strain Pv 4-95 was chosen to study the effect of AdpA on the production of secondary metabolites. First, we performed a phylogenetic analysis of this strain. The phylogenetic analysis based on the sequence of the 16S rRNA gene using the RDP Classifier program showed that the Pv 4-95 strain belongs to the genus *Streptomyces*. BLAST analysis of the 16S rRNA gene sequence of this strain demonstrated the highest similarity with the *S. platensis* strain ATCC23948 (99.86% identity). The NJ phylogenetic tree that included strains Pv 4-95, the four closest related *Streptomyces* species, and several representative *Streptomyces*-type strains also confirmed the affiliation with the genus *Streptomyces* (Figure 1 and Figure S1).

The heterologous *adpA* gene was introduced into the Pv 4-95 strain to activate the synthesis of secondary metabolites, which primarily exhibit antimicrobial activity. As a result, we obtained the exconjugate strain Pv 4-95 containing the recombinant plasmid pTESadpA. To compare the metabolic profiles of both strains, the secondary metabolite extracts were analysed by LC–MS using the wild-type strain Pv 4-95 and Pv 4-95pTES, which contained the empty pTES vector as a control. The obtained strains were cultivated in SG medium, and the secondary metabolites were extracted, dissolved in methanol and measured by LC-MS.

**Figure 2:**
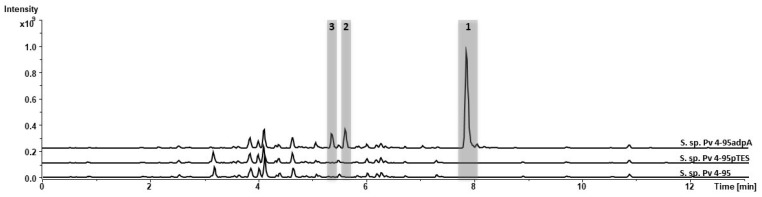
Chromatograms of the crude extracts from the *Streptomyces* sp. Pv 4-95adpA, which contains the adpA gene, the *Streptomyces* sp. Pv 4-95pTES harbouring the empty pTES vector, and the strain *Streptomyces* sp. Pv 4-95 (wild type).

A comparative analysis of the obtained chromatograms of Pv 4-95adpA and the control strains Pv 4-95pTES and Pv 4-95 revealed three new mass peaks with UV maxima at λ_max_ 226 and 326 nm eluting at retention times of 7.8 min for peak 1 (*m*/*z* 209.163 [M+H]^+^), 5.6 min for peak 2 (*m*/*z* 225.159 [M+H]^+^) and 5.4 min for peak 3 (*m*/*z* 207.148 [M+H]^+^ (Figure 2 and Figure S2). The calculated monoisotipic masses and UV spectra of these compounds were compared with entities from the Dictionary of Natural Products Database (DNP) [26]. The database search revealed several hits for diketopiperazine compounds from fungal sources, so we decided to purify the compounds to determine their structures by NMR spectroscopy.

### 3.2. Purification and Structure Elucidation of the Activated Compounds

**Figure 3:**
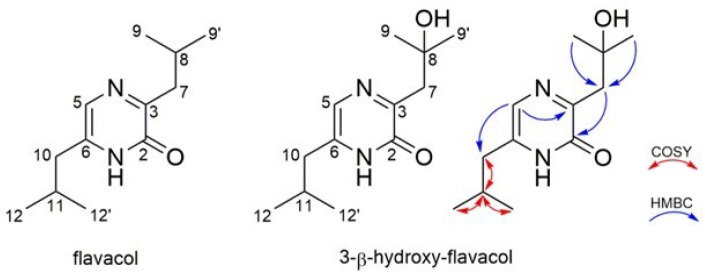
Structure of flavacol and 3-*β*-hydroxy-flavacol with the key HMBC and COSY correlations.

The *Streptomyces* sp. Pv 4-95adpA strain was cultured in 10 L of SG medium, and the secondary metabolites were extracted with ethyl acetate. Three purification steps yielded 2.9 and 1.2 mg of Compounds **1** (*m*/*z* 209.163 [M+H]^+^) and **2** (*m*/*z* 225.159 [M+H]^+^), respectively. Compound **3** (*m*/*z* 207.148 [M+H]^+^) could not be obtained by our purification approach. The molecular formula of Compound **1** was determined to be C_12_H_20_N_2_O based on the monoisotopic mass of 208.158 *m*/*z*. Structural analysis by NMR identified Compound **1** as flavacol (Figure 3, Table 1).

Compound **2** showed a molecular formula of C_12_H_20_N_2_O_2_ based on the monoisotopic mass 224.152 *m*/*z*. The mass difference indicated a derivative of flavacol that contained a hydroxyl group. The structure was assigned by 1D and 2D NMR experiments (Figures S3–S9), and the hydroxyl group was determined to be at the isobutyl moiety at position 3, resulting in the new derivative 3-*β*-hydroxy flavacol (Figure 3, Table 1).

Flavacol is a colourless crystalline metabolite of *Aspergillus flavus* that has been described by Dunn et al. [27]. Micetich and MacDonald [28] showed that flavacol is an intermediate product of the synthesis of neoaspartic acid and neohydroxyaspartic acid in *Aspergillus* species and is a condensed product of leucine and isoleucine. Flavacol has previously been identified as a secondary metabolite of the fungi *Aspergillus* spp. And *Penicillium* spp. [27,28,29,30] and bacteria [31] and has shown inhibitory activity on the mammalian mitochondrial respiratory chain [32].

The presence of the heterologous *adpA* gene in the *Streptomyces* sp. Pv4-95 strain resulted in the activation of flavacol synthesis and its novel derivative 3-*β*-hydroxy flavacol. Only small amounts of pyrazines are produced by bacteria, despite their wide detection range [33]. Pyrazines are 1,4-dinitrogen-substituted benzenes that are widely used in food, agriculture and medicine. These compounds are common in plants, insects, fungi and bacteria [34]. The pleiotropic regulator AdpA in streptomycete strains can positively and negatively influence BGC expression [11] or activate silent BGCs [12]. This could radically alter the metabolic profile of these strains. In our study, the presence of this global regulator led to the synthesis of compounds from the pyrazine group. Most researchers working with pyrazines consider that their synthesis is not enzymatic. In turn, they suggest that pyrazines are formed by the nonenzymatic condensation of amino acids [35]. Thus, we hypothesize that heterologous AdpA affects the biosynthesis of precursors by increasing their number, which is then condensed into the resulting compounds.

## 4. Conclusions

In summary, we identified the pyrazine compound flavacol and its new derivative 3-*β*-hydroxy flavacol by integrating the pleiotropic transcriptional regulator AdpA in *Streptomyces* sp. Pv 4-95 isolated from the rhizosphere soil of *P. viridiglaucescens*. Thus, heterologous expression of *adpA* gene in actinobacteria should help to explore their biosynthetic potential via the activation of cryptic natural product gene clusters. The *Streptomyces* sp. Pv 4-95 (collection number Lv 740) and *Streptomyces* sp. Pv 4-95adpA, which contains the heterologus *adpA* gene (collection number Lv 741), were deposited in the CCMPA of LNU.

## Figures and Tables

**Table 1:** NMR data of 3-*β*-hydroxy flavacol and flavacol (500 MHz in MeOD-*d4*).

3-*β*-Hydroxy-Flavacol (2)			Flavacol (1)		
Atom #	*δ_C_*, mult.	*Δ_H_*, mult. (*J* in Hz)	Atom #	*δ_C_*, mult.	*Δ_H_*, mult. (*J* in Hz)
1	NH	-	1	NH	-
2	159.8, C	-	2	159.9, C	-
3	155.8, C	-	3	158.0, C	-
4	N	-	4	N	-
5	124.0, CH	7.2, s	5	123.5, CH	7.1, s
6	141.0, C	-	6	140.4, C	-
7	46.3, CH_2_	3.0, s	7	42.6, CH_2_	2.6, d (7.25)
8	72.6, C	-	8	28.2, CH	2.2, m
9/9′	29.8, CH_3_	1.2, s	9/9′	23.1, CH_3_	0.9, d (6.62)
10	40.2, CH_2_	2.4, d (7.35)	10	40.2, CH_2_	2.4, d (7.25)
11	29.7, CH	2.0, m	11	29.6-, CH	2.0, m
12/12′	22.5, CH_3_	1.0, d (6.65)	12/12′	22.5, CH_3_	1.0, d (6.62)

## Data Availability

Not applicable.

## Supplementary Materials

The following supporting information can be downloaded at: https://www.mdpi.com/article/10.3390/microorganisms10122335/s1, Figure S1: Partial sequence of the *Streptomyces* sp. Pv4-95 16S ribosomal RNA gene; Figure S2: Analyses of identified peaks in the crude extract of *Streptomyces* sp. Pv 4-95adpA strain. (A) Mass spectrum and (B) UV spectrum of identified peaks corresponding to flavacol (1), 3-β-hydroxy flavacol (2) and a compound that could not be obtained (3); Figure S3: ^1^H NMR spectrum (MeOD-*d4*, 500 MHz) of flavacol (1); Figure S4: ^13^C NMR spectrum (MeOD-*d4*, 125 MHz) of flavacol (1); Figure S5: ^1^H NMR spectrum (MeOD-*d4*, 500 MHz) of 3-*β*-hydroxy flavacol (2); Figure S6: ^13^C NMR spectrum (MeOD-*d4*, 500 MHz) of 3-*β*-hydroxy flavacol (2); Figure S7: ^1^H-^1^H COSY spectrum (MeOD-*d4*, 500 MHz) of 3-*β*-hydroxy flavacol (2); Figure S8: Edited-HSQC spectrum (MeOD-*d4*, 500 MHz) of 3-*β*-hydroxy flavacol (2); Figure S9: HMBC spectrum (MeOD-*d4*, 500 MHz) of 3-*β*-hydroxy flavacol (2).
