# Supplementary material for: Flavacol and Its Novel Derivative 3-β-Hydroxy Flavacol from Streptomyces sp. Pv 4-95 after the Expression of Heterologous AdpA
Source: Microorganisms. 2022 Nov 25;10(12):2335. doi: 10.3390/microorganisms10122335 (PMC9783318; doi:10.3390/microorganisms10122335)
Supplement: Supplementary file 1 [file microorganisms-10-02335-s001.zip › microorganisms-2054922-supplementary.pdf]

Supplementary Material

Flavacol and its Novel Derivative 3-*β*-hydroxy Flavacol from *Streptomyces* sp. Pv 4-95 after the Expression of Heterologous AdpA

Stepan Tistechok[1], Marc Stierhof[2], Anna Kachor[1,3], Maksym Myronovskyi[2], Oleksandr Gromyko[1,4*] and Andriy Luzhetskyy[2, 5*]

[1] Department of Genetics and Biotechnology, Ivan Franko National University of Lviv, 79005 Lviv, Ukraine; steptistechok@gmail.com (S.T.); anya.aiva18@gmail.com (A.K.)

[2] Department of Pharmaceutical Biotechnology, Saarland University, 66123 Saarbruecken, Germany; m.stierhof@t-online.de (M.S.); maksym.myronovskyi@uni-saarland.de (M.M.)

[3] Explogen LLC, 79005 Lviv, Ukraine;

[4] Microbial Culture Collection of Antibiotic Producers, Ivan Franko National University of Lviv, 79005 Lviv, Ukraine;

[5] Helmholtz Institute for Pharmaceutical Research Saarland, 66123 Saarbruecken, Germany;

*Correspondence: a.luzhetskyy@mx.uni-saarland.de; Tel.: +49-681-302-70200 (A.L.)
oleksandr.gromyko@lnu.edu.ua; Tel.: +38-032-23-94-407 (O.G.);

Physical data of 3-β-hydroxy flavacol (2): white solid; 1.2 mg; UV (31% ACN in H_2O + 0.1% FA) λ_{max} (log ε) 226 nm (1.92) and 326 nm (1.73); ^1H and ^{13}C NMR data, see Table 1; ESI-TOF-MS *m/z* 225.15903 $[M+H]^+$ (calc. for $C_{12}H_{20}N_2O_2$ 224.1519).

```
>OM763959.1 Streptomyces sp. strain Pv4-95 16S ribosomal RNA gene, partial
sequence
TGCAAGTCGAACGATGAACCTCCTTCGGGAGGGGATTAGTGGCGAACGGGTGAGTAACACGTGGGCAATC
TGCCCTTCACTCTGGGACAAGCCCTGGAAACGGGGTCTAATACCGGATACGACTACCGACCGCATGGTCT
GGTGGTGGAAAGCTCCGGCGGTGAAGGATGAGCCCGCGGCCTATCAGCTTGTTGGTGGGGTGATGGCCTA
CCAAGGCGACGACGGGTAGCCGGCCTGAGAGGGCGACCGGCCACACTGGGACTGAGACACGGCCCAGACT
CCTACGGGAGGCAGCAGTGGGGAATATTGCACAATGGGCGAAAGCCTGATGCAGCGACGCCGCGTGAGGG
ATGACGGCCTTCGGGTTGTAAACCTCTTTCAGCAGGGAAGAAGCGAAAGTGACGGTACCTGCAGAAGAAG
CGCCGGCTAACTACGTGCCAGCAGCCGCGGTAATACGTAGGGCGCAAGCGTTGTCCGGAATTATTGGGCG
TAAAGAGCTCGTAGGCGGCTTGTCACGTCGGATGTGAAAGCCCGGGGCTTAACCCCGGGTCTGCATTCGA
TACGGGCAGGCTAGAGTTCGGTAGGGGAGATCGGAATTCCTGGTGTAGCGGTGAAATGCGCAGATATCAG
GAGGAACACCGGTGGCGAAGGCGGATCTCTGGGCCGATACTGACGCTGAGGAGCGAAAGCGTGGGGAGCG
AACAGGATTAGATACCCTGGTAGTCCACGCCGTAAACGTTGGGAACTAGGTGTGGGCGACATTCCACGTC
GTCCGTGCCGCAGCTAACGCATTAAGTTCCCCGCCTGGGGAGTACGGCCGCAAGGCTAAAACTCAAAGGA
ATTGACGGGGGCCCGCACAAGCAGCGGAGCATGTGGCTTAATTCGACGCAACGCGAAGAACCTTACCAAG
GCTTGACATACACCGGAAACGCCTGGAGACAGGCGCCCCCTTGTGGTCGGTGTACAGGTGGTGCATGGCT
GTCGTCAGCTCGTGTCGTGAGATGTTGGGTTAAGTCCCGCAACGAGCGCAACCCTTGTTCTGTGTTGCCA
GCATGCCCTTCGGGGTGATGGGGACTCACAGGAGACTGCCGGGGTCAACTCGGAGGAAGGTGGGGACGAC
GTCAAGTCATCATGCCCCTTATGTCTTGGGCTGCACACGTGCTACAATGGCCGGTACAATGAGCTGCGAT
ACCGCGAGGTGGAGCGAATCTCAAAAAGCCGGTCTCAGTTCGGATTGGGGTCTGCAACTCGACCCCATGA
AGTCGGAGTTGCTAGTAATCGCAGATCAGCATTGCTGCGGTGAATACGTTCCCGGGCCTTGTACACACCG
CCCGTCACGTCACGAAAGTCGGTAACACCCGAAGCCGGTGGCCCAACCCCTTGTGGGAGGGAATCGTCGA
```

Figure S1. Partial sequence of the *Streptomyces* sp. Pv4-95 16S ribosomal RNA gene.

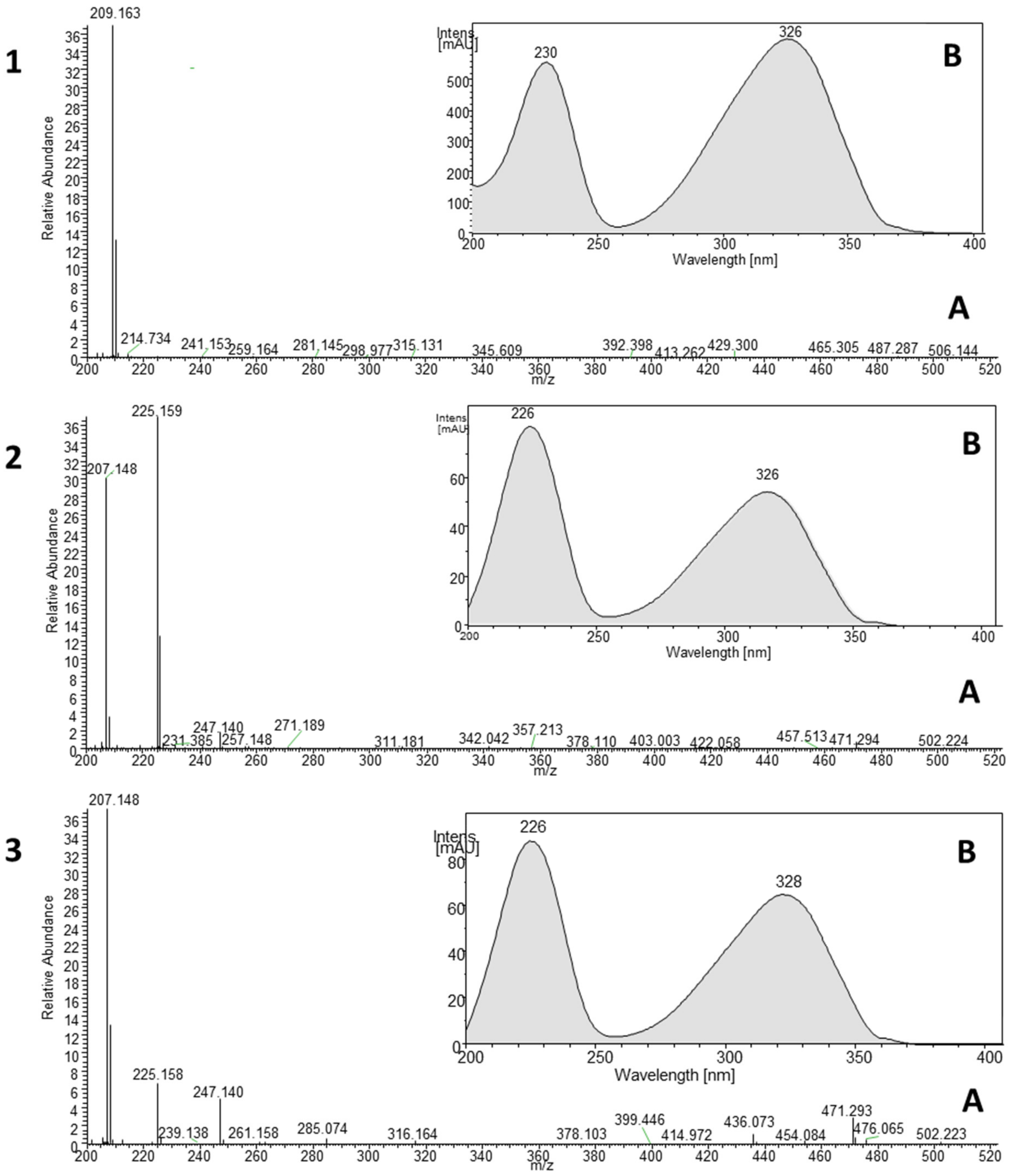

Figure S2. Analyses of identified peaks in the crude extract of *Streptomyces* sp. Pv 4-95adpA strain. (A) Mass spectrum and (B) UV spectrum of identified peaks corresponding to flavacol (1), 3-β-hydroxy flavacol (2) and a compound that could not be obtained (3).

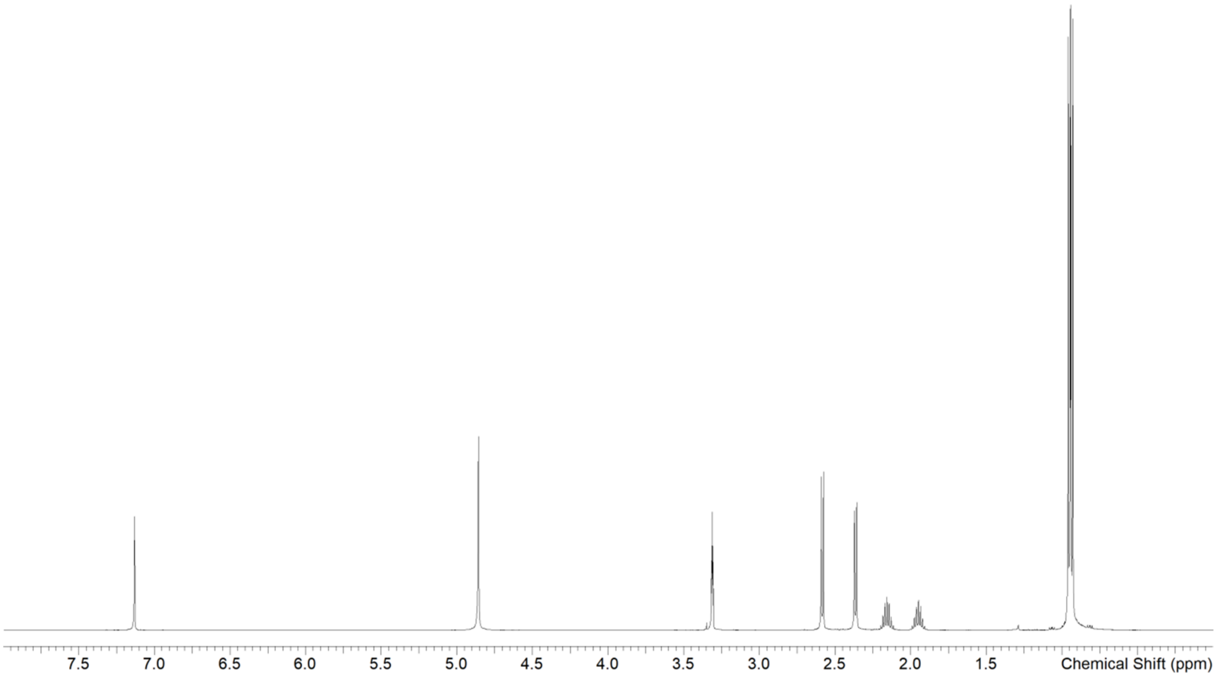

Figure S3. ^{1}H NMR spectrum (MeOD-*d4*, 500 MHz) of flavacol (1).

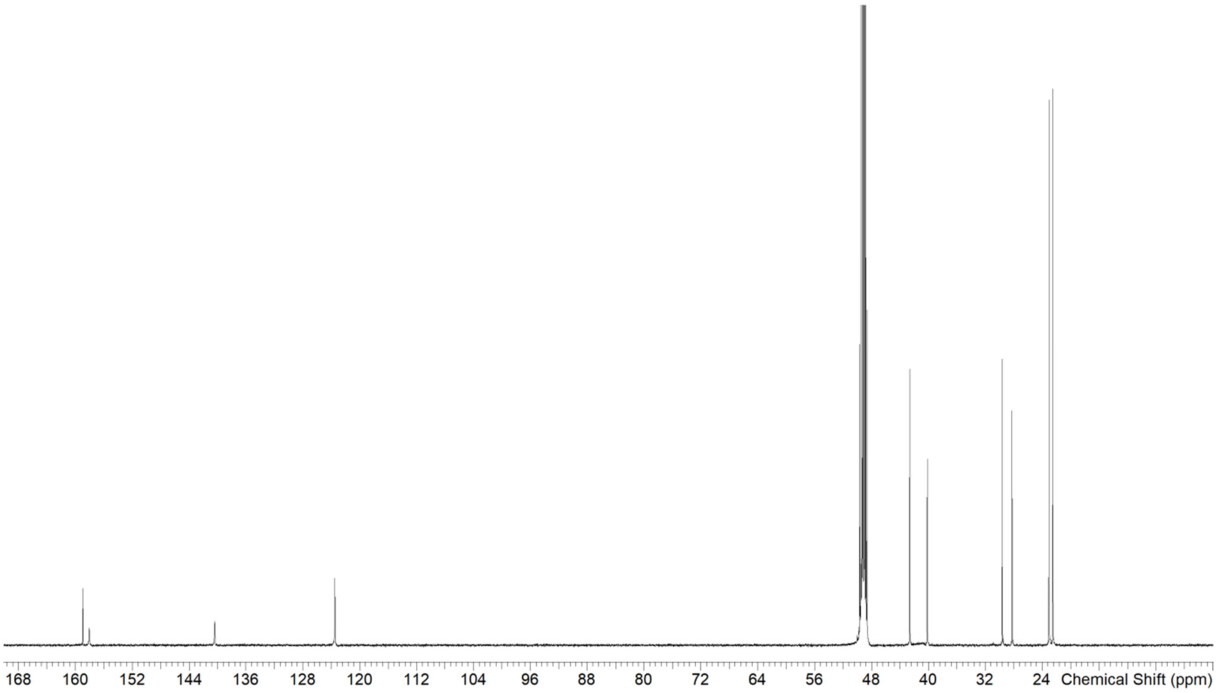

Figure S4. ^{13}C NMR spectrum (MeOD-*d4*, 125 MHz) of flavacol (1).

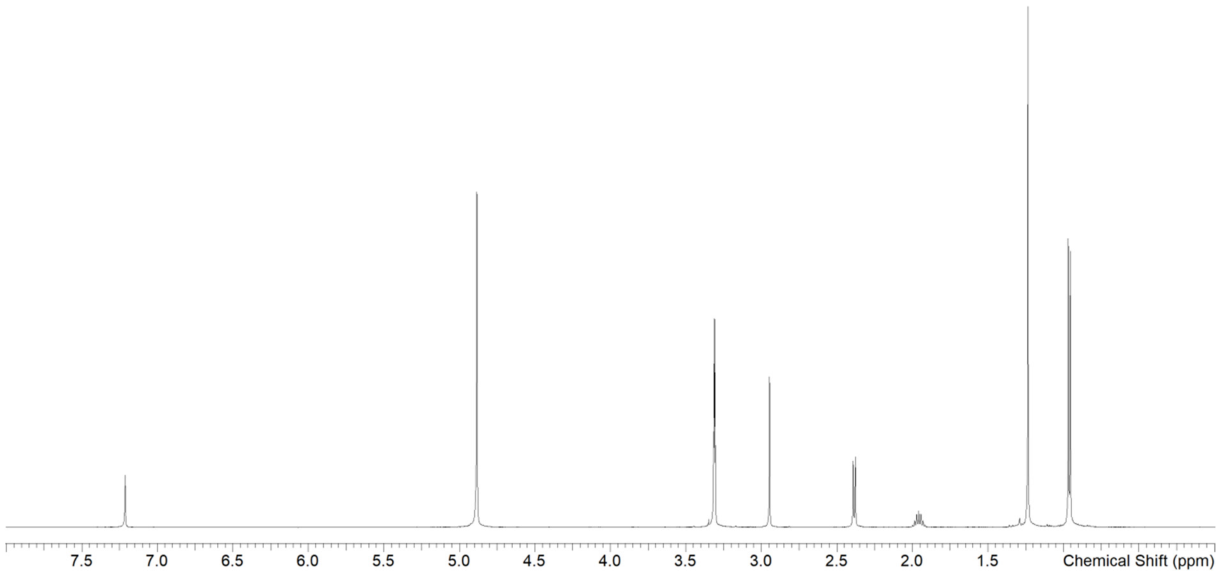

Figure S5. ^{1}H NMR spectrum (MeOD-*d4*, 500 MHz) of 3-*β*-hydroxy flavacol (2).

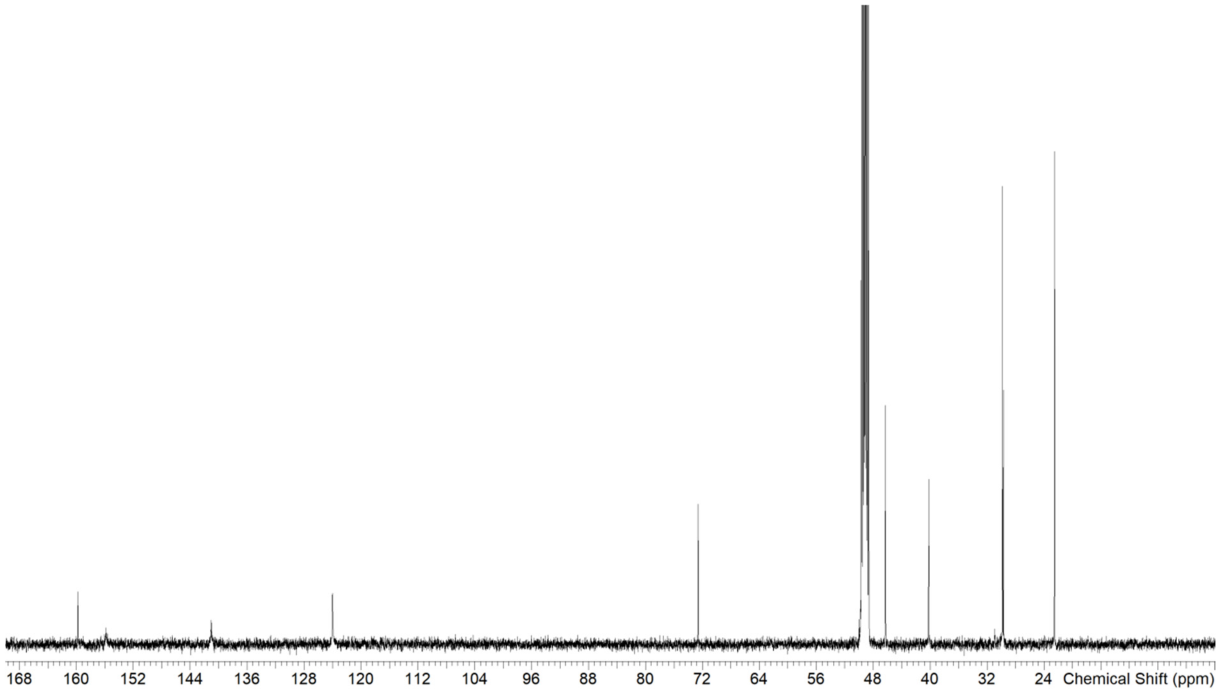

Figure S6. ^{13}C NMR spectrum (MeOD-*d4*, 500 MHz) of 3-*β*-hydroxy flavacol (2).

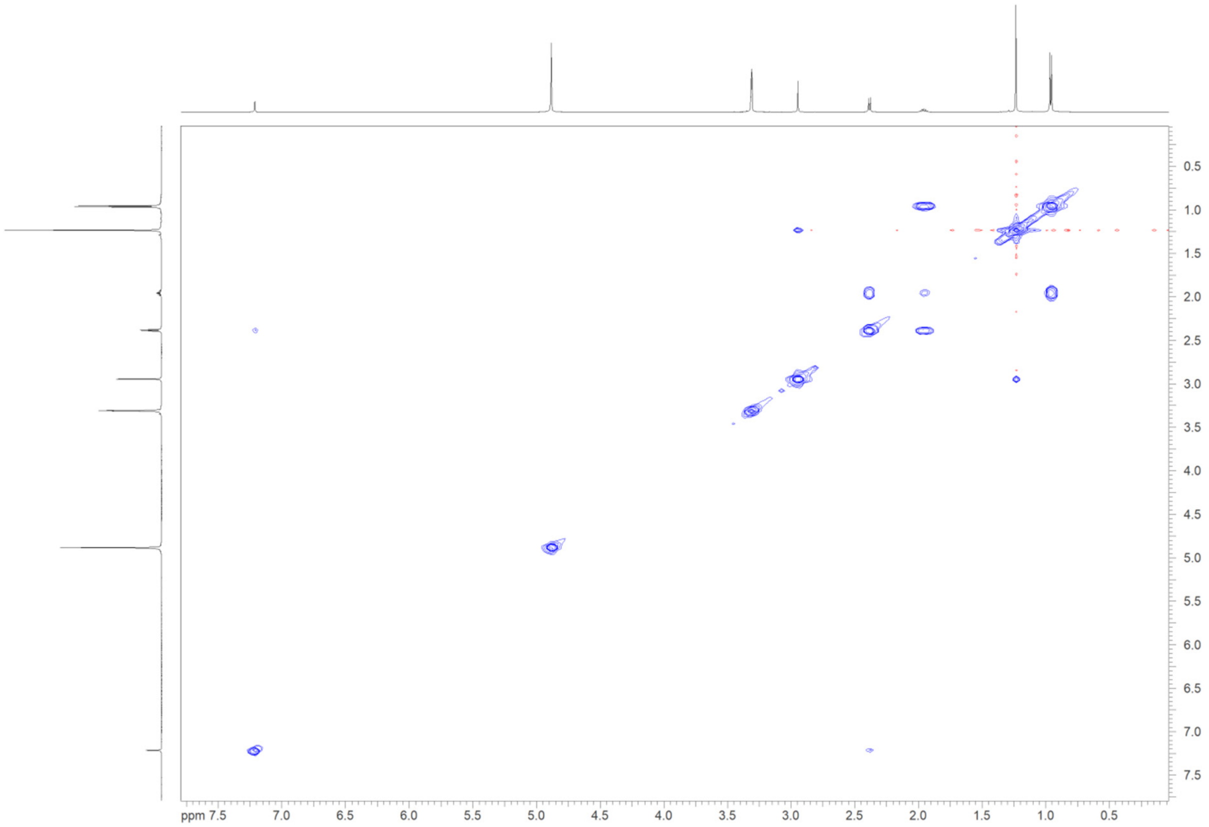

Figure S7. ^{1}H-^{1}H COSY spectrum (MeOD-*d4*, 500 MHz) of 3-*β*-hydroxy flavacol (2).

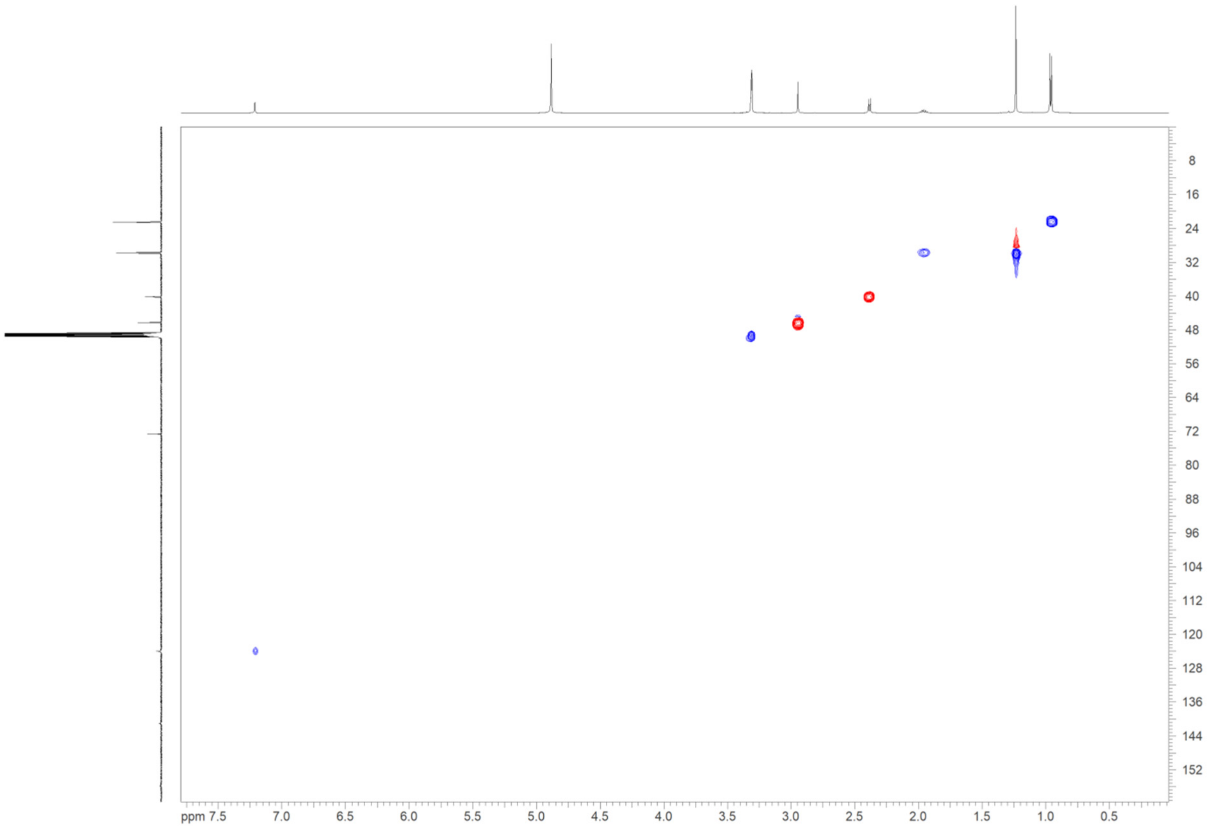

Figure S8. Edited-HSQC spectrum (MeOD-*d4*, 500 MHz) of 3-*β*-hydroxy flavacol (2).

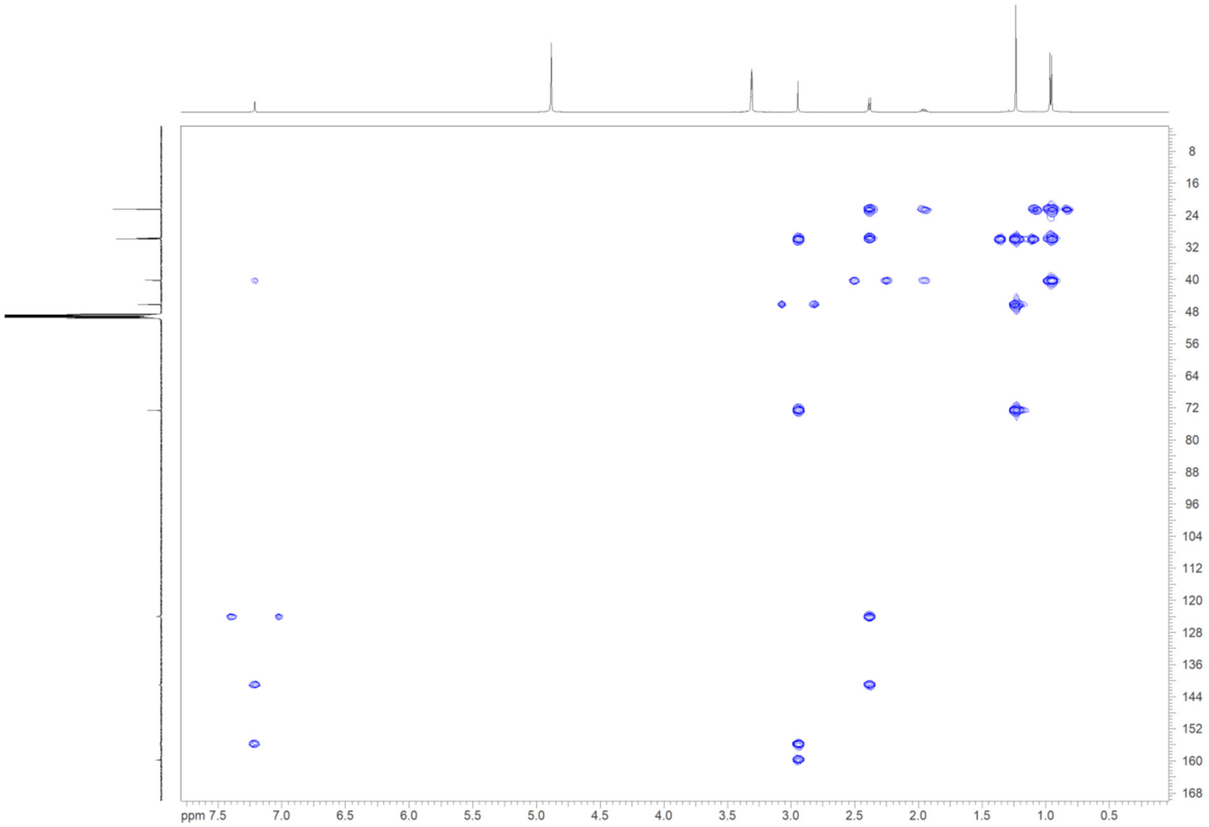

Figure S9. HMBC spectrum (MeOD-*d4*, 500 MHz) of 3-*β*-hydroxy flavacol (2).
